# Unraveling neuronal and metabolic alterations in neurofibromatosis type 1

**DOI:** 10.1186/s11689-024-09565-6

**Published:** 2024-08-31

**Authors:** Valentina Botero, Seth M. Tomchik

**Affiliations:** 1 Department of Neuroscience and Pharmacology, University of Iowa, Iowa City, IA USA; 2 Stead Family Department of Pediatrics, University of Iowa, Iowa City, IA 52242 USA; 3 Iowa Neuroscience Institute, University of Iowa, Iowa City, IA 52242 USA; 4 Fraternal Order of Eagles Diabetes Research Center, University of Iowa, Iowa City, IA 52242 USA; 5 Hawk-IDDRC, University of Iowa, Iowa City, IA 52242 USA; 6 Department of Neuroscience, Scripps Research, Scripps Florida, Jupiter, FL USA; 7 Skaggs School of Chemical and Biological Sciences, Scripps Research, La Jolla, CA USA

**Keywords:** Neurofibromatosis type 1, Neurofibromin, NF1, Metabolism

## Abstract

Neurofibromatosis type 1 (OMIM 162200) affects ~ 1 in 3,000 individuals worldwide and is one of the most common monogenetic neurogenetic disorders that impacts brain function. The disorder affects various organ systems, including the central nervous system, resulting in a spectrum of clinical manifestations. Significant progress has been made in understanding the disorder’s pathophysiology, yet gaps persist in understanding how the complex signaling and systemic interactions affect the disorder. Two features of the disorder are alterations in neuronal function and metabolism, and emerging evidence suggests a potential relationship between them. This review summarizes neurofibromatosis type 1 features and recent research findings on disease mechanisms, with an emphasis on neuronal and metabolic features.

## Introduction

Neurofibromatosis type 1 (NF1) is a multisystemic autosomal-dominant condition affecting ~ 1 in 3,000 live births worldwide [1–4]. Historical recognition of the disorder dates to ancient Egypt [5, 6], with Friedrich Daniel von Recklinghausen providing the first comprehensive clinical description in 1882. Initially termed “von Recklinghausen disease,” it is now known as neurofibromatosis type 1 [7, 8]. Significant progress has been made in understanding NF1’s genetic underpinnings. The disorder is caused by mutations in a single gene, neurofibromin 1 (*NF1*) [9], which encodes the protein neurofibromin (Nf1). Several pivotal discoveries made in the late 1990s include the establishment of diagnostic criteria for accurate NF1 diagnosis [10, 11], mapping of the *NF1* gene to chromosome 17q11.2 [12–14], and the identification of its protein product [12, 15–19]. Despite these advances (and more since then), gaps persist in our understanding of the disorder and its underlying genetic, cellular, and systemic mechanisms. Features of the disorder include alterations in neuronal and brain function as well as metabolic alterations [20–24]. Some of the features of the disorder, including the brain/cognitive symptoms, could be influenced by the metabolic alterations. Here we review the mechanisms of NF1 pathophysiology, with a focus on the emerging understanding of neuronal and metabolic alterations.

## Diagnostic criteria and clinical features

**Fig. 1:**
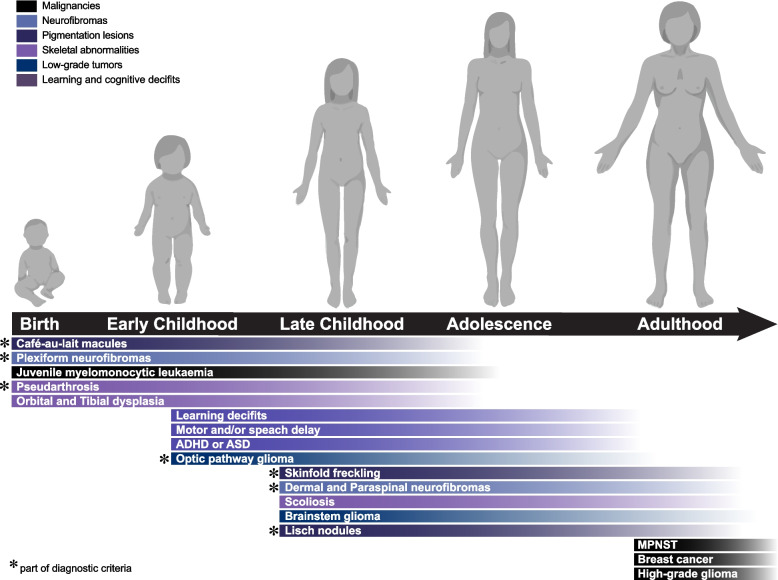
Disease progression and clinical features of NF1. The onset and severity of NF1 clinical features vary between individuals. In children, the most common clinical physical manifestations are skeletal abnormalities such as scoliosis, tibial dysplasia, and café-au-lait spots. Young children are at risk of developing juvenile myelomonocytic leukemia, optic gliomas, and behavioral and cognitive deficits, with attention-deficit/hyperactivity disorder (ADHD) and autism spectrum disorder (ASD) being the most common. The risk of developing plexiform neurofibromas (pNF) is high during the early stages of life, but other malignancies such as malignant peripheral nerve sheath tumors (MPNST) and breast cancer occur more often after the third decade of life [25, 26]. Created with BioRender.com

NF1 is characterized by a broad spectrum of clinical manifestations that begin in infancy and progressively worsen (Fig. 1) [25, 26]. Diagnostic criteria for NF1, first established in 1987 [10] and updated in 2021 [11] (Table 1), rely on a physical examination and family history review. Some symptoms emerge in an age-dependent manner, making proper diagnosis during early childhood challenging, particularly for those lacking a family history of the disease (half of NF1 cases stem from de novo mutations) [3, 11, 20]. These challenges led to revisions of the diagnostic criteria, incorporating mosaic neurofibromatosis and genetic testing [11]. Symptoms of NF1 include increased susceptibility to various tumors, including peripheral nerve tumors like neurofibromas, plexiform neurofibromas, and malignant peripheral nerve sheath tumors, as well as brain tumors such as optic pathway gliomas and brainstem gliomas [1, 25, 27, 28]. Although tumors are a primary clinical characteristic of NF1, it also produces non-tumor symptoms including pigmentation defects, skeletal abnormalities, stunted growth, cognitive impairments, and behavioral alterations [27, 29]. NF1 reduces life expectancy by 8–15 years [30–32] and significantly impacts quality of life, with up to 80% of children experiencing moderate to severe cognitive impairments [22, 29].

**Table 1:** Diagnostic criteria for NF1

Diagnostic Criteria
**Individual presents with two**^**a**^** or more of the following:**
1. Six or more café-au-lait macules of ≥ 5 mm in diameter before puberty or ≥ 15 mm in diameter after puberty
2. Axillary or inguinal freckling
3. Two or more dermal neurofibromas or one plexiform neurofibroma
4. An optic pathway glioma
5. Two or more iris Lisch nodules or choroidal abnormalities
6. A distinctive osseous lesion such as a sphenoid dysplasia, anterolateral bowing of the tibia, or pseudoarthritis of a long bone
7. A heterozygous NF1 variant fraction of 50% in apparently normal tissue such white blood cells

^a^A child of a parent with NF1 merits diagnosis if one or more of the features are present

## The *NF1* gene and neurofibromin protein

NF1 results from mutations in the *NF1* gene, which encodes a large 2,818 amino acid protein called neurofibromin (Nf1) [17, 33]. The Nf1 protein contains a central Ras-GTPase activating protein (GAP)-related domain (GRD) [16, 34]. It primarily localizes to the cytoplasm, interacting with Ras at the plasma membrane, and is also found in the nucleus, endoplasmic reticulum, and mitochondria [35–38]. Nf1 is ubiquitously expressed throughout development, with the highest levels in nervous system cells, including Schwann cells, neurons, astrocytes, and oligodendrocytes [17, 19, 39]. Clinical manifestations of NF1 are variable, even among identical germline *NF1* mutations, and some exhibit segmental or mosaic NF1.

### Role of the NF1 gene and neurofibromin GAP-related domain

**Fig. 2:**
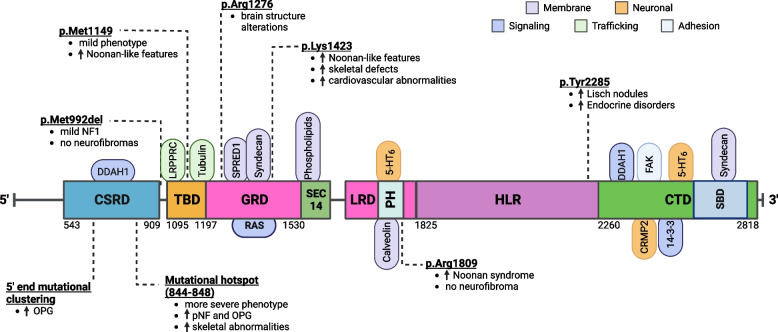
Nf1 protein structure, interacting domains, and genotype–phenotype correlations. Nf1 protein contains several domains (squares) and interacting proteins (ovals). Nf1 protein domains include the following: cysteine-serine-rich domain (CSRD), tubulin binding domain (TBD), central GTPase-activating-protein-related domain (GRD), SEC14 domain, leucine-rich domain (LRD), pleckstrin homolog (PH), HEAT-like repeats (HLR), C-terminal domain (CTD), syndecan-binding domain (SBD). Phospholipids and proteins identified as Nf1-interacting proteins are shown in association with their described function, such as: trafficking (green), neuronal (yellow), membrane localization (purple), cell adhesion (gray), and cell signaling (blue). Nf1 mutations reported to correlate with certain phenotypes are shown above/below the protein and associated phenotypes. Numbers along protein indicate amino acid residues. Figure adapted and modified from Ratner and Miller (2015) [26], Mo et al. (2022) [59], and Anastasaki et al. (2022) [60]. Created with BioRender.com

Over 2,600 unique mutations within the *NF1* gene have been identified [40] (Fig. 2). Clinical heterogeneity of NF1 can be attributed to multiple factors, including allelic variation, second-hit mutations, epigenetic changes, differences across the *NF1* mutations, and tissue-specific Nf1 functions [41]. Nf1 protein expression across between different mutations (i.e., Nf1 heterozygosity can result in ~ 12–89% of normal protein expression level) [42]. Neurofibromas result from loss of *NF1* heterozygosity following second-hit mutations [43]. Other symptoms of the disorder emanate from haploinsufficiency due to the heterozygous mutation itself. Heterozygous germline mutation in *NF1* is associated with notable impacts on cognitive functions, affecting attention and learning and increasing the prevalence of autism spectrum disorder (ASD) [44, 45]. Among the *NF1* isoforms, certain variants have a tissue-specific role. The alternatively-spliced exon 11alt12 (formerly known exon 9a) is predominantly expressed in the central nervous system (CNS), particularly within forebrain neurons [46]. In contrast, the alternatively spliced exon 30alt31 (formerly known as exon 23a) contains an alternative exon that lies within the GRD and diminishes Ras GAP activity [47]; mice lacking this exon have learning and communication impairments but are not susceptible to tumor formation [48–50]. Alternative splicing at the 3’ end of *NF1* produces the alternative exon 56alt57 (formerly 48a) which is expressed highly in fetal and adult cardiac and skeletal tissue and may contribute to reduced muscle strength and muscle weight [47, 51–54]. Exon 12alt13 (formerly known as 10a-2) is a low-level ubiquitous isoform concentrated in perinuclear granular structures [55]. Moreover, the *NF1*-∆E43 isoform shows elevated expression in the liver, kidneys, lungs, placenta, and skeletal muscle relative to the general expression of *NF1* [56, 57]. Dimerization of the Nf1 protein and the varied impacts of different mutations on protein stability further complicate the disease’s pathophysiology [58].

### Neurofibromin’s impact on cellular processes via Ras and cAMP signaling

**Fig. 3:**
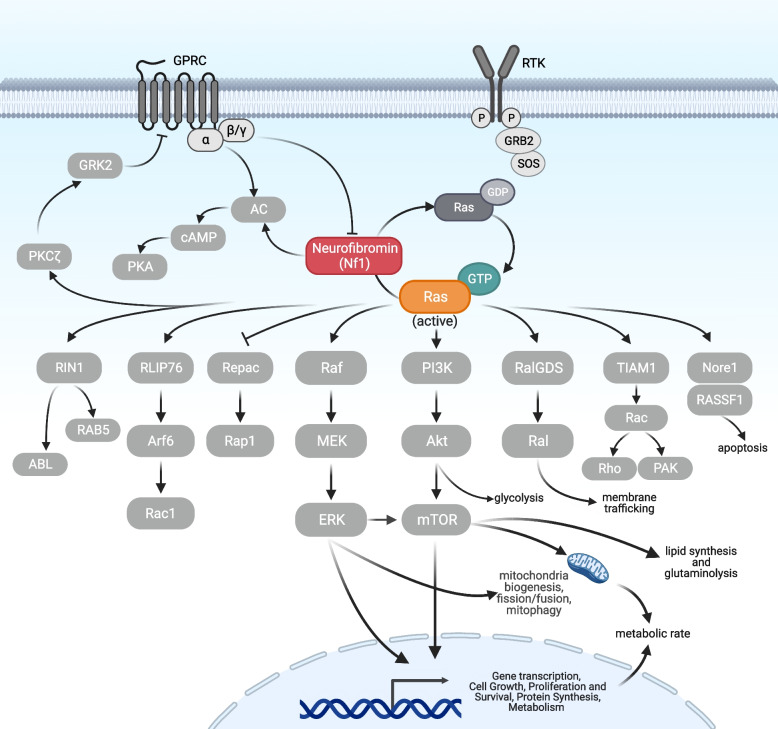
Nf1 regulates molecular functions in key biological signaling pathways via Ras. Nf1 affects diverse cellular functions by regulating several signaling pathways. Nf1 accelerates the conversion of active GTP-bound Ras into inactive GFP-bound Ras, thereby regulating numerous downstream effectors. Known signaling pathways downstream of Nf1 and Ras signaling include the Raf/MEK/ERK and PI3K/AKT/mTOR pathways. Figure created with BioRender.com and adapted from Anastasaki et al. (2022) [60], Masgras and Rasola (2021) [66], and Ratner and Miller (2015) [26]

The Nf1 protein features multiple structural domains, with the GRD being the most extensively studied [16, 59–62]. The GRD plays a pivotal role in the regulation of Ras signaling, catalyzing hydrolysis of Ras-bound GTP into GDP and thereby attenuating Ras signaling (Fig. 3). Consequently, *NF1* loss-of-function mutations lead to the accumulation of active Ras-GTP and aberrant activation of downstream pathways, including Raf/MEK/ERK and PI3K/AKT/mTOR [45, 61, 63, 64]. Numerous mutations that compromise the function of the GRD have been identified in patients (Fig. 3) [61]. Analysis of the crystallographic structure of the Nf1 GRD revealed a critical arginine finger residue (R1276) that stabilizes and positions Ras association with the catalytic domain. Notably, a patient mutation (R1276P), which substitutes arginine with proline, results in a > 1000-fold reduction in Ras-GAP activity [61, 62, 65].

Nf1 regulates multiple cellular processes, including metabolism, cell proliferation, differentiation, and survival via its regulatory effects on Ras signaling. Nf1/Ras activity is regulated by upstream signal transduction pathways involving receptor tyrosine kinases (RTK). One such RTK is the Anaplastic Lymphoma Kinase (ALK), which interacts with Nf1 and functions as an upstream activator of Nf1-regulated Ras signaling pathway [67–70]. In addition to RTKs, other upstream regulators of Nf1/Ras include G protein-coupled receptors (GPCRs), specifically the Gβγ subunits that bind to Nf1 in striatal neurons and inhibits its capacity to suppress Ras/AKT/mTOR signaling [71].

Loss-of-function mutations in Nf1 dysregulate multiple signaling pathways downstream of Ras, including the canonical mitogen-activated protein kinase (MAPK) signaling pathway (Raf/MEK/ERK), PI3K/AKT/mTOR, and others (Fig. 3) [18, 63, 64]. These pathways in turn regulate multiple cellular and metabolic processes, including cell growth, survival, nutrient uptake, proliferation, and the modulation of neuronal metabolism in response to growth factors, nutrients, and changes in the cellular energy state [72]. Hyperactivation of Raf/MEK/ERK due to loss of Nf1 is one of the major mechanisms implicated in NF1 phenotypes and is a current therapeutic target [73–75]. Conditional Nf1 knockout in various brain cells (astrocytes, pyramidal cells, GABAergic neurons, and inhibitory/excitatory neurons) increases ERK signaling [37]. In addition, hyperactivation of the PI3K/AKT/mTOR pathway contributes to the development of some NF1-associated phenotypes. The convergence of these two Ras effector pathways, each of which will be discussed in greater detail below, underscores the complexity of cellular signaling alterations in NF1. Overall, the NF1 gene and the Nf1 GRD function as a central regulator of Ras signaling, modulating downstream signaling targets and affecting diverse cellular functions.

In addition to its role in regulating Ras signaling, Nf1 is a positive regulator of cyclic adenosine monophosphate (cAMP) and downstream protein kinase A (PKA) activity (Fig. 3). Nf1 is required for normal cAMP generation in neurons and astrocytes, as observed in *Drosophila* and rodent models of NF1 [76, 77]. In turn, alterations in cAMP/PKA signaling are implicated in many of the NF1 phenotypes, including cell differentiation/growth and learning [68, 78–81]. Furthermore, NF1-related changes in cAMP/PKA levels are altered via a non-canonical mechanism involving Ras-dependent phosphorylation of protein kinase C zeta (PKCζ) [82, 83]. This pathway regulates neuronal cAMP homeostasis in both human-induced pluripotent stem cell (hiPSC)-derived neurons and primary mouse neuron cultures [82].

## Mechanisms of pathophysiology in neurofibromatosis type 1

### Tumors

Tumor formation is a primary concern in NF1, with cutaneous neurofibromas (CN) and plexiform neurofibromas (pNF) pervasive among patients. CNs, affecting over 99% of NF1-afflicted adults, are benign but prolific tumors that emerge during late childhood and experience rapid grow during puberty and pregnancy [4]. The number of CNs in adults can reach into the thousands, leading to significant disfigurement and considerable physical and psychological distress [84, 85]. Conversely, pNFs affect about 50% of patients, proliferating rapidly during childhood and adolescence [86]. These tumors are located in peripheral nerve sheaths, with Schwann cells representing the major neoplastic cell type [87]. Neurofibromas can cause pain, disfigurement, and impair neurovascular structures and airways. Notably, pNFs are major contributors to the elevated mortality rates in NF1, as they can transform into malignant peripheral nerve sheath tumors (MPNST), which have a low survival rate [31, 88, 89]. Although surgical intervention is the standard treatment for pNF, it is typically palliative [88]. In 2020, selumetinib, a MEK inhibitor, gained United States Food and Drug Administration (FDA) approval for treating symptomatic and inoperable pNF in children [73–75].

In addition to neurofibromas, optic pathway gliomas (OPGs) are the second most common tumors in NF1—approximately 15–20% of children with NF1 develop OPGs [90]. Although OPGs are often non-lethal, about 30% of affected individuals will experience visual decline or loss due to these tumors, significantly reducing their quality of life. Given the severe delayed toxicity of radiotherapy and increased risk of visual loss with surgery, chemotherapy is the first line of treatment for OPGs that cause visual decline. Notably, females are three times more likely to require treatment [90–95].

Experimental animal models of NF1 and in vitro cellular studies have provided significant insights into NF1-related tumor development. These models show significant alterations in growth, cell proliferation, and tumor progression. Such phenotypes stem from the interplay between multiple tissues, signaling pathways, neurite growth, and neuronal excitability. Introducing patient-derived NF1 mutations into hiPSCs impairs Schwann cell differentiation, promotes stemness, and fosters neurofibroma formation [96]. Furthermore, studies using hiPSCs and murine models have revealed that Nf1 mutations increase neuronal excitability, exacerbating tumor progression in both the central and peripheral nervous system [97–99]. Neuronal activity and midkine expression directly impact the development and progression of mouse *Nf1*-OPG [97, 98]. Importantly, the progression of optic glioma growth in *Nf1* mutant mice can be selectively suppressed with clinically relevant dosing of lamotrigine, an anti-epileptic drug, for months after treatment cessation [100].

Aberrant regulation of the Raf/MEK/ERK signaling pathway plays a pivotal role of NF1-related tumorigenesis [26, 101], and MEK is a therapeutic target [73–75, 102, 103]. Both human and mouse models of MPNST exhibit aberrant activation of ERK (one molecular step downstream of MEK). Targeted pharmacological inhibition of the Raf/MEK/ERK pathway has been shown to inhibit tumor progression [101]. Proteomic analysis has also revealed Ras/PI3K-dependent activation of mTOR signaling in astrocytes from human or mutant mice optic nerve gliomas [63]. In NF1-deficient cells and human tumors, mTOR is constitutively activated [63, 64]. Notably, pharmacological inhibition of mTOR, MEK, and AKT signaling can restore normal proliferation in *Nf1*-deficient astrocytes [63, 104]. Additional pathways, such as cAMP/PKA, are also targets of interest for therapeutic interventions, playing roles in regulating cell differentiation and growth arrest [78]. In *Drosophila,* Nf1 regulates growth through non-cell-autonomous control of cAMP/PKA signaling in neuroendocrine cells [68]. In human neural progenitor cells, loss of Nf1 decreases cAMP levels, resulting in smaller growth cone areas and shorter axonal lengths [82]. These neural deficits can be restored through increased cAMP levels and by inhibiting Ras activity [82].

The zebrafish model, known for its transparent embryos, offers a unique lens through which to study the role of Nf1 during development. The zebrafish genome contains two *NF1* orthologs, *nf1a* and *nf1b,* each with over 90% similarity to human *NF1* at the amino acid level [105]. Experiments involving transient knockdown of these *nf1* orthologs during embryogenesis result in vascular patterning defects, echoing observations seen in murine NF1 models and mirroring hallmarks of the human disease [105]. Furthermore, *nf1a* and *nf1b* zebrafish larvae exhibit hyperplasia of oligodendrocyte progenitor and Schwann cells [106, 107]. Additionally, *nf1* knockout initiates gliomagenesis in adult zebrafish brain tissue [108].

*NF1* mutations introduced into Yucatan miniature pigs (minipigs) mimic characteristics commonly observed in NF1 patients. Two mutations have been introduced, which model prevalent human *NF1* mutations: *NF1*^*R1947X*^*,* representing a common nonsense mutation, and *NF1*^+*/ex42del*^, emulating a heterozygous *NF1* mutation [109, 110]. Minipigs with either of these *NF1* mutations exhibit major clinical hallmarks of NF1, including café-au-lait macules (CALMs), OPGs, and neurofibromas [109, 110]. Notably, the minipig is unique among model organisms in that it exhibits spontaneous loss of NF1 heterozygosity, which drives tumor formation in humans [109].

### Behavioral deficits and neuronal alterations

Cognitive impairment is a prevalent complication of NF1, affecting approximately 80% of those diagnosed with NF1 [22, 111]. Individuals with NF1 are significantly more likely to encounter a spectrum of developmental delays, such as deficits in learning, memory, executive function, broad language deficits, and fine motor skills [22, 29, 112]. NF1 patients may exhibit below-average IQ scores, with a small subset (4–8%) falling into the intellectually impaired range [22, 113]. The disorder is also highly comorbid with attention-deficit/hyperactivity disorder (ADHD) and ASD. Approximately half of the children with NF1 are diagnosed with ADHD [22, 111], and 12–49% exhibit symptoms of ASD [114–118]. These cognitive and behavioral challenges significantly impact quality of life of NF1 patients, affecting their emotional well-being, physical health, role functioning, and social interactions [25].

Given that NF1 increases risk for cognitive/behavioral symptoms, a major question is how loss of neurofibromin affects neuronal/brain function. Studies utilizing various animal models, including flies, zebrafish, mice, and minipigs, have contributed to understanding the role of Nf1 function in the nervous system. The *Drosophila* Nf1 protein, sharing 60% amino acid sequence homology with its human counterpart and conserved Ras GAP functionality [80], serves as an outstanding model for investigating genetics, neuronal function, and molecular signaling pathways in vivo [119–121]. In *Drosophila,* Nf1 is ubiquitously expressed during development and is prominently localized in the adult nervous system [39]. Loss of Nf1 function in flies disrupts sleep and circadian rhythms [122–124]; the circadian rhythm deficit can be rescued by restoring the expression of wild-type *Drosophila* Nf1 in neurons or by attenuating Ras/ERK signaling pathways [122]. Additionally, *Drosophila* Nf1 mutants exhibit learning and memory deficits, including impaired olfactory associative learning and deficits in short-, middle-, and long-term memory [67, 79, 125, 126]. These learning and memory impairments can be rescued by restoring wild-type Nf1 protein in a neuron-specific manner [125] and ameliorated by enhancing PKA activity [79, 80]. Additionally, pharmacologically and genetically attenuating ALK, an upstream RTK, rescues associative learning deficits in *nf1* mutants [67].

Mutations in the *Drosophila NF1* ortholog increase locomotor activity and spontaneous grooming [127, 128], phenotypic analogs of the ADHD symptoms common in NF1 patients [29, 111]. Nf1/Ras signaling regulates grooming behavior, as the Nf1 GRD is required in neurons to maintain normal levels of grooming in *Drosophila* [127]. Besides motor-related behaviors, *Drosophila nf1* mutants display social and behavioral alterations, including delayed flight and climbing responses and altered sleep patterns [80, 122, 129]. Loss of Nf1 alters social behavior, specifically male courtship [130]. Synaptic transmission at the neuromuscular junction is altered in *nf1* mutants, suggesting that synaptic physiology changes may contribute to the phenotypes [131, 132].

Murine models of NF1 have been invaluable in unraveling Nf1's functions within the nervous system via structural plasticity and modulation of signaling pathways. In the rat hippocampus, the loss of Nf1 function disrupts pyramidal dendritic spine structural plasticity, resulting in the activity-dependent loss of dendritic spines due to sustained Ras activation [133]. In mice, Nf1 haploinsufficiency (*Nf1*^±^) replicate cognitive and behavioral deficits observed in NF1 patients, manifesting as deficits in hippocampal spatial learning and reduced long-term potentiation driven by increased GABA-mediated inhibition [44, 45, 48]. Rescue of Raf/MEK/ERK activity, either pharmacologically or genetically, ameliorates learning deficits and rescues long-term potentiation [44, 134, 135]. Moreover, *Nf1*^±^ mice show heightened excitability in sensory neurons. Along with dopamine deficiency, this could contribute to learning impairment [99, 136–138]. Additionally, both human *NF1* and mouse *Nf1* are enriched in inhibitory neurons within the cortex [139]. Nf1 plays a crucial role in the nervous system beyond cognition and physiology, as human-derived *Nf1* mutations increase neuronal excitability in mice, accelerating tumor progression in the central and peripheral nervous system [97, 98].

Several other vertebrate models like zebrafish and minipigs have recapitulated neurocognitive deficits similar to those observed in NF1 patients. Zebrafish with nf1 mutations display learning and memory deficits, including short- and long-term habituation; these can be restored either through pharmacological inhibition of Ras downstream targets or by increasing cAMP signaling [81, 107]. The *NF1*^+*/ex42del*^ mutation in the minipig model produces neurocognitive deficits akin to those observed in NF1 patients, including learning and memory impairments and hyperactivity [110]. In addition, the NF1 minipig model exhibits altered pain sensitivity associated with NF1—examination of dorsal root ganglia expressing mutant *NF1*^+*/ex42del*^ revealed dysregulation of calcium and sodium channels [110]. Overall, these findings underscore the importance of Nf1 function in regulating neuronal development, structure, activity, and function.

### Metabolic alterations

**Fig. 4:**
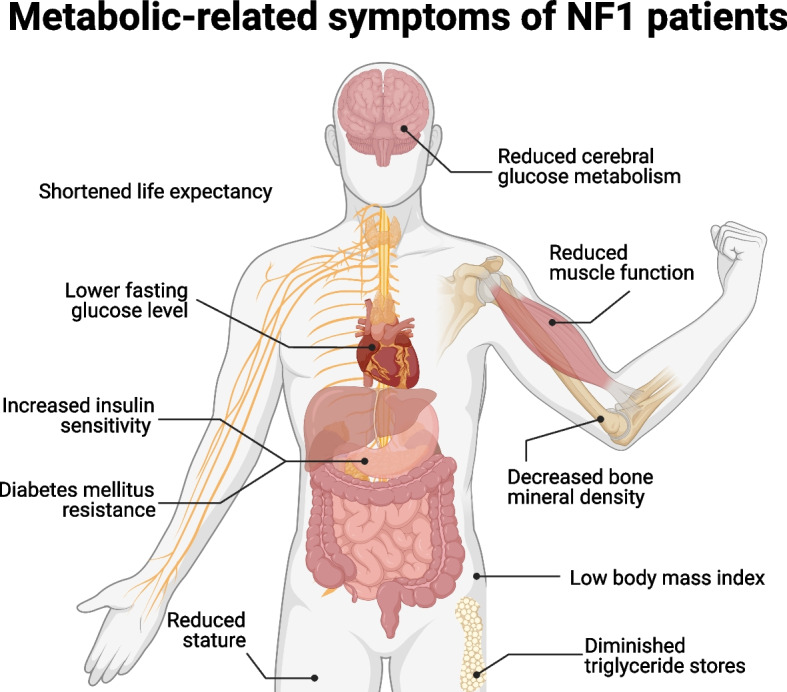
Neurofibromatosis type 1 metabolic-related symptoms. Multisystemic alterations in metabolism that are commonly associated with NF1. Modified from Masgras and Rasola (2021) [66]. Created with BioRender.com

Metabolism is altered in multiple ways in NF1, and these changes may contribute to the pathophysiology of the disease. Patients with NF1 exhibit systemic metabolic shifts (Fig. 4) [32, 140]. Studies on body composition reveal multiple anomalies, including a lower body mass index (BMI) [141], reduced triglyceride stores [142], decreased bone mineral density [143], and shorter stature relative to unaffected individuals [144]. NF1 patients display lower muscle function [141], reduced maximal muscular strength [24, 145], and compromised motor proficiency [146]. In a comprehensive analysis of resting energy expenditure (REE), women with NF1 display heightened REE despite lower BMI [24]. Additionally, NF1 patients have a lower respiratory quotient (RQ), which indicates a differential reliance on fat oxidation over carbohydrate metabolism [24].

In addition to altered body composition, individuals with NF1 present a metabolic profile characterized by lower fasting blood glucose levels [147], heightened insulin sensitivity [148], and a reduced incidence of diabetes mellitus [140, 149, 150]. Hormonal dysregulation in NF1 patients, involving alterations in leptin, vistafin, and adiponectin, may contribute to these metabolic features [148]. Also noted are decreased levels of calcium, calcitonin, and vitamin D [143]. In adults with NF1, there is a notable reduction in cerebral glucose metabolism in the thalamus, as evidenced by positron emission tomography scans [23]. In addition to these differences, individuals with NF1 often experience significant cognitive and physiological fatigue [151], suggesting that metabolic dysregulation may impact brain function.

The Ras/Raf/MEK/ERK pathway regulates multiple metabolic processes, including cell proliferation, protein synthesis, lipid and cholesterol homeostasis, adipocyte differentiation, lipolysis, lipogenesis, gluconeogenesis, and gene expression (Fig. 3) [152, 153]. Consequently, hyperactivation of this pathway due to loss of Nf1 may mediate the observed cellular and systemic metabolic dysfunctions. Nf1-deficient cells exhibit increased glycolysis and reduced mitochondrial respiration mediated through the Ras/MEK/ERK pathway [154].

**Fig. 5:**
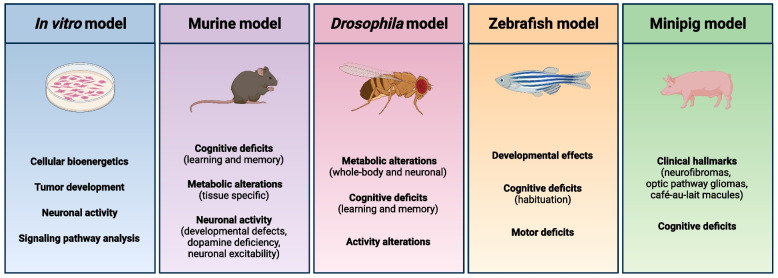
Animal and in vitro models of NF1. Strengths of in vitro, murine, *Drosophila*, zebrafish, and minipig models to investigate NF1. Created with BioRender.com

Metabolic features of the disorder have been recapitulated in animal models of NF1 (Fig. 5). Heterozygous *Nf1* (*Nf1*^±^) mice exhibit altered body composition, represented by a reduction in fat mass and increased percentage of lean mass [155]. Similar to NF1 patients, the loss of Nf1 function enhances insulin sensitivity and glucose utilization in *Nf1*^±^ mice [148, 155]. Conditional *Nf1* knockout results in metabolic changes in muscles, including reduced muscle growth, increased triglyceride content, malformations (cardiac, renal, hepatic, and skeletal muscle defects), and prenatal lethality [155–157]. Inactivation of *Nf1* in skeletal muscle (*Nf1*_*MyoD*_^*−/−*^) proves lethal within the first week of life; during development, animals with the mutation exhibit stunted growth and intramyocellular lipid accumulation, indicative of impaired long chain fatty acid metabolism [156, 158]. Notably, muscle samples from limb-specific *Nf1* conditional knockout (*Nf1*_*Prx1*_^*−/−*^) mice recapitulate some of the pathological findings observed in human NF1 muscle biopsies, including intramyocellular lipid accumulation, elevated oxidative metabolic enzyme activity, heightened expression of leptin and fatty acid synthase, and reduced fatty acid transporters [156, 158].

Muscle weakness in NF1 may stem from changes in lipid storage resembling lipid storage myopathies. Nf1 manipulations in mice suggest a role for Nf1 in metabolic regulation within muscle tissue, suggesting avenues for potential therapeutic interventions. For example, when Nf1 is lost in mesenchymal tissues (*Nf1*_*Prx1*_^*−/−*^ mice), dietary interventions that reduce long chain fatty acid intake and enrich medium-fatty acids with L-carnitine effectively rescue lipid accumulation and muscle weakness [158]. Additionally, pharmacological intervention using the selective MEK inhibitor, PD98059, rescues postnatal body weight loss and lipid accumulation in mice with muscle-specific *Nf1* knockout when administered during pregnancy in *Nf1*_*MyoD*_^*−/−*^ dams [159]. In pediatric NF1 patients, pharmacological inhibition of MEK with selumetinib or PD0325901 has led to clinically significant improvements in muscular strength [74], supporting a MEK/ERK-dependent mechanism underlying Nf1-associated muscle metabolism. Lastly, recent studies have highlighted the cell-autonomous role of Nf1 in postnatal muscle growth and metabolic homeostasis, with homozygous *Nf1* mutations resulting in neonatal lethality [160]. Overall, these data suggest a critical role for Nf1 in muscle development and function, providing insights into potential therapeutic interventions.

Research involving several models suggests that the metabolic alterations associated with NF1 extend beyond muscle tissue. In *Drosophila,* loss of Nf1 drives multiple phenotypes indicative of metabolic dysfunction. Nf1 mutations decrease body size by 15–25%, mirroring the short stature observed in NF1 patients [68, 80, 141, 161]. Restoring human or *Drosophila* Nf1 expression in *nf1* mutant neurons can rescue the mutant growth defect [77, 161, 162]. Additionally, *Drosophila nf1* mutants experience a significant reduction in lifespan due to altered mitochondrial respiration and increased production of reactive oxygen species (ROS) [163]. Overexpressing Nf1 in *nf1* mutants rescues lifespan, enhances mitochondrial respiration, and significantly reduces ROS production [163]. Furthermore, Nf1 regulates metabolic homeostasis, with Nf1 deficiency increasing metabolic rate (CO_2_ production and O_2_ consumption), decreasing glycogen and triglyceride stores, and increasing the rate of lipid turnover [164, 165]. Similar to human NF1 patients, Nf1 knockdown in *Drosophila* results in a reduced RQ [164], indicating increased fat utilization. These effects emanate, at least in part, from neuronal mechanisms [164]. Additionally, loss of Nf1 heightens starvation susceptibility and increases feeding, likely in a compensatory manner to the metabolic alterations [164]. The metabolic phenotype in *Drosophila* resembles the increased REE observed in NF1 patients [24], suggesting metabolic dysfunction across different species (and cell types). Notably, the Nf1 metabolic and motor (grooming) phenotypes are caused by the loss of Nf1 function in different neural subsets, as knockdown in metabolism-regulating neurons does not affect grooming [164]. Collectively, these metabolic alterations highlight Nf1's tight regulation of metabolic function and its susceptibility to disruption when Nf1 is lost.

The molecular mechanism of Nf1’s effects on metabolism involves its activity as a Ras GAP. The catalytic activity within the GRD of Nf1 is required for Nf1-dependent modulation of metabolic rate. A patient-derived mutation in the GRD (R1320P) fails to rescue metabolic phenotypes [164]. This mutation is at the equivalent residue as R1276P in humans, which reduces Ras-GAP activity by over 1000-fold without impairing any other Nf1 function [61]. Transgenic expression of full-length, wild-type Nf1 selectively in neurons of *nf1* mutants restores normal metabolic function [164] (as well as grooming activity) [127]. Additionally, the activation of downstream targets of Nf1 and Ras, such as ERK, play a significant role in driving metabolic effects. Constitutive ERK activation in metabolic-regulating neurons increases metabolic rate, phenocopying the metabolic dysregulation observed in Nf1 mutations [164]. Beyond neuronal and muscle-specific effects, metabolic profiling of mouse embryonic fibroblasts (MEFs) derived from Nf1 knockout animals has revealed significant alterations in cellular bioenergetics. Specifically, Nf1 knockout MEFs displayed diminished mitochondrial activity, driven by elevated glycolysis and decreased respiration [154]. These metabolic shifts stem from heightened Ras/MEK/ERK signaling within mitochondria [154]. Collectively, these results suggest that Nf1 acts in neurons, muscles, and potentially additional cell types to regulate cellular bioenergetics in multiple model systems. Further, the mechanism involves the overactivation of Ras/Raf/MEK/ERK activity. The contributions of other downstream signaling pathways (e.g., the metabolism-regulating mTOR pathway) provide potentially promising avenues for exploration in the study of metabolic regulation in NF1.

### Pigmentary lesions

Pigmentary features are crucial for early diagnosis of NF1. CALMs, which are observed in 99% of NF1 patients by age 1 [166], consist of melanocytes with biallelic *NF1* inactivation [10, 167]. These features are among the earlies signs of the disease. Additionally, axillary and inguinal freckling, typically appear between 3 to 5 years of age and are present in about 90% of patients by age 7 [166]. Another significant marker are Lisch nodules, which are asymptomatic hyperpigmented iris hamartomas, typically appear by age 5–6. These nodules are present in over 70% of patients by the age of 10 and are observed in over 90% of adults with NF1 [166, 168]. Two *NF1* mutations in minipig models, *NF1*^*R1947X*^ and *NF1*^+*/ex42del*^, have successfully replicated these pigmentary features, including CALMs and axillary freckling [109, 110]. The loss of Nf1 expression in minipigs models of NF1 results in hyperactivation of the Ras pathway and its effector molecules, linking the signaling cascades to the pathogenesis of cutaneous NF1 features [110]. Finally, homozygous *nf1a* and *nf1b* mutant zebrafish larvae exhibit pigmentation anomalies, providing a novel vertebrate model to study pigmentation lesions associated with NF1 [107].

### Skeletal abnormalities

Patients with NF1 exhibit a range of skeletal abnormalities, leading to significant morbidity. These osseous defects include both localized and generalized bone deformities, contributing to bone weakening and an increased fracture risk. One of the most significant manifestations is long-bone dysplasia, which affects approximately 5% of individuals with NF1. This condition is characterized by anterolateral bowing of the lower limbs, predominately affecting the tibia, resulting in decreased bone density, increased fracture risk, and pseudarthrosis [11, 92, 169, 170]. Another notable skeletal anomaly is sphenoid-wing dysplasia, which affects up to 11% of NF1 patients and results in distinct cranial deformities [88, 171, 172]. Scoliosis is the most prevalent skeletal defect associated with NF1, occurring in up to 30% of patients and often necessitating surgical intervention in severe cases [173]. Furthermore, NF1 patients tend to be shorter than their healthy counterparts, with 8–15% experiencing a generalized reduction in skeletal bone growth [174, 175]. Individuals with NF1 exhibit both local and general dysregulation of bone resorption and remodeling, leading to increased formation of osteoclast [176, 177]. NF1 patients often have a reduced bone mineral density, osteoporosis, and increased risk of bone fractures [178].

In animal models, Nf1 is critical in skeletal development. In mice, Nf1 is essential for joint development; conditional Nf1 loss during early limb development induces multiple joint abnormalities, including deformities in the hip, knee, and elbow [179]. Similar to human NF1 patients, tibia bowing occurs in mice due to Nf1 deficiency, leading to growth retardation and abnormal growth plate development [179]. Moreover, the expression of Nf1 in bone marrow osteoprogenitors is crucial for maintaining adult skeletal integrity [180]. Loss of Nf1 in these cells leads to skeletal anomalies resembling those seen in NF1 patients, including progressive scoliosis, kyphosis, tibial bowing, and deformities in the skull and anterior chest wall [181]. Additionally, Nf1 loss in osteochondroprogenitors results in decreased bone mass, increased cortical porosity, severe short stature, and intervertebral disc defects [181]. Similar skeletal phenotypes are observed in a minipig model of NF1. The *NF1*^+*/ex42del*^ minipig model develops tibial bone curvature and shorter long bones such as the femur, tibia, humerus, ulna, metacarpals, indicative of reduced stature [182]. These animal models further substantiate the critical role of Nf1 in skeletal integrity and development.

### Developmental alterations

The loss of Nf1 results in developmental alterations that may contribute to the clinical manifestations of NF1. Magnetic resonance imaging studies have documented alterations in neuronal development among NF1 patients, including increased total brain and white matter volumes. Notably, enlargements in subregions including corpus callosum and brainstem, as well as increased optic nerve tortuosity, are commonly observed [20, 183–196]. Along with structural differences, loss of Nf1 is linked to a range of functional changes in neuronal activity. These include changes in cortical association networks and functional connectivity within the default network, corticostriatal functional circuits, and areas critical for cognition, social functioning, executive functioning, and spatial working memory [197–205]. Collectively, these observations underscore the role of Nf1 in modulating brain development, connectivity, and function.

Conditional knockout of neuronal Nf1 in mice mirrors human pathology by enlarging the corpus callosum, an effect which can be rescued by inhibiting Raf/MEK/ERK signaling during neonatal development [206]. In a more severe manifestation, homozygous *Nf1* knockout (*Nf1*^*−/−*^*)* mice exhibit gestational lethality due to severe cardiovascular abnormalities, highlighting a significant role for Nf1 during tissue development [157]. Heterozygous *Nf1* mutant (*Nf1*^±^) mice, although viable, exhibit numerous brain abnormalities, including enlarged glia, increased neuron numbers, astrocyte proliferation, and neural tube closure defects [157, 207–209]. Neuron-specific *Nf1* knockout in mice also reveals brain abnormalities, including abnormal cortex development, increased cell density, heightened astrocyte proliferation, and reduced cortical thickness [210]. Loss of Nf1 function in neurons, rather than glia, in mice causes growth defects, further underscoring Nf1’s critical role in neuronal growth and development [210, 211].

The structural alterations resulting from the loss of Nf1 raise significant questions about whether and how these developmental alterations influence behavior. Understanding this relationship is crucial for optimizing the timing of therapeutic interventions by, for instance, allowing for targeted treatments during appropriate developmental times. While direct behavioral correlations in humans are yet to be established, animal models have provided valuable insights. For example, in *Drosophila*, the developmental contribution of Nf1 to adult behavior has been parsed. Loss of Nf1 increases the frequency of spontaneous grooming behavior in adult animals [128]. Additionally, studies using conditional knockdown of Nf1 in neurons across developmental time windows revealed that loss of Nf1 during a critical developmental period impairs motor (grooming) behavior, whereas similar alterations either earlier (embryonic stage) or later (adult stage) do not have the same effect [127]. The mechanisms by which Nf1 loss impacts neuronal development in NF1 are diverse and complex. They may include altered cell growth, division, differentiation/specification, apoptosis, dendrite & axon targeting, synaptogenesis, activity-dependent synaptic refinement, hormone responsivity, and nutrient responsivity [210–213]. Future mechanistic studies are necessary to dissect how developmental disruptions due to the loss of Nf1 result in adult phenotypes.

## Conclusions

Research utilizing animal models and in vitro studies has elucidated the significant effects of Nf1 in the nervous system and behavior, identifying its significance in normal development and function. Nf1 influences cellular and systemic physiology via multiple molecular and cellular mechanisms, including alterations in metabolism. Several major models, such as mice, *Drosophila,* minipigs, and zebrafish, have considerably advanced our understanding of Nf1's mechanistic role within the nervous system and its effects on metabolic regulation. Continued advancements in these areas hold promise for the development of novel targeted therapies and interventions aimed at improving the outcomes and quality of life for individuals with NF1.

## Data Availability

N/A (Review).

## Abbreviations

ADHD: Attention-deficit/hyperactivity disorder
ALK: Anaplastic Lymphoma Kinase
ASD: Autism spectrum disorder
BMI: Body mass index
CALM: Café-au-lait macule
cAMP: Cyclic adenosine monophosphate
CN: Cutaneous neurofibromas
CNS: Central nervous system
FDA: Food and Drug Administration
GAP: GTPase activating protein
GPCR: G protein-coupled receptor
GRD: GAP-related domain
hiPSC: Human induced pluripotent stem cells
MAPK: Mitogen-activated protein kinase
MEF: Mouse embryonic fibroblasts
MPNST: Malignant peripheral nerve sheath tumors
NF1: Neurofibromatosis type 1
*NF1*: Human neurofibromin 1 gene
*Nf1*: Non-human neurofibromin 1 gene
Nf1: Neurofibromin protein
*nf1*: Invertebrate mutant neurofibromin protein
OPG: Optic pathway glioma
PKA: Protein kinase A
PKCζ: Protein kinase C zeta
pNF: Plexiform neurofibroma
REE: Resting energy expenditure
ROS: Reactive oxygen species
RQ: Respiratory quotient
RTK: Receptor tyrosine kinase
